# Applying a multi-layered, mixed methods approach to evaluate technology and workforce interventions in Kenyan neonatal units

**DOI:** 10.1080/16549716.2025.2558267

**Published:** 2025-09-25

**Authors:** Michuki Maina, Sassy Molyneux, Fred Were, Dorothy Oluoch, Edna Mutua, David Gathara, Mike English, Abdulazeez Imam, Gloria Ngaiza, Asma Rababeh, Gulraj Grewal, Naima Nasir, Caroline Waithira, Nancy Odinga, Vincent Kagonya, Onesmus Onyango, Kenneth Karumba, Caroline Jones, Peter Mwangi, Lucy Kinyua, Lydia Thuranira, Virginia Njoroge, Ngina Mwangi, Loise Mwangi, Penina Musyoka, Zainab Kioni, Sebastian Suarez Fuller

**Affiliations:** a KEMRI-Wellcome Trust Research Programme, Health Services Research Group, Nairobi, Kenya; b Nuffield Department of Medicine, University of Oxford, Oxford, UK; c Kenya Paediatric Research Consortium, Nairobi, Kenya; d Department of Health, County Government of Nyeri, Nyeri, Kenya; e Department of Health, County Government of Kiambu, Kiambu, Kenya; f Department of Health, County Government of Embu, Embu, Kenya; g Department of Health, County Government of Machakos, Machakos, Kenya

**Keywords:** Ward assistants, neonatal care, health workforce, quality of care, participatory research design

## Abstract

Understanding how to best implement healthcare innovations in resource-poor settings requires complex interventions and evaluations, often straining existing healthcare systems. We aimed to address this challenge in our Kenyan neonatal intervention research by deeply integrating stakeholder engagement, applying layered methodologies, and leveraging our research team’s diverse expertise. This paper reports on the design and implementation of a research programme examining the impact of new staff introductions on care quality, staff, and family experiences within an existing neonatal technology programme in Kenya. Collaborating with stakeholders, our multidisciplinary programme of research included the analysis of routine health data before and after staff introductions, co-creation of novel quantitative data collection tools, and embedded qualitative research. Continuous feedback from participants, stakeholders, and researchers facilitated an adaptive approach, with timelines and methods adjusted to minimise participant burden. This iterative process allowed for robust data collection to inform health system improvements. Our findings provide insights into implementation and evaluation research for healthcare innovations in resource-constrained settings and emphasise ethical, context-sensitive research practices for sustainable health system strengthening.

## Paper context

- **Key findings:** The paper reports the use of layered mixed methodologies to conduct implementation research of a complex intervention in Kenyan neonatal hospitals
- **Added knowledge:** This paper provides a detailed approach to designing and implementing a complex health intervention in settings that are under resourced. Sustained stakeholder engagement throughout the conceptualisation, design, and implementation of research is essential for both intervention success and knowledge generation.
- **Global health impact for policy and action**: This study highlights the need for researchers to engage policy makers and stakeholders to support research on complex interventions and evaluations and ethical research practices.

## Introduction

It is increasingly recognised that while technologies have the potential to improve clinical care, the complexity of healthcare settings needs consideration for these improvements to be realised [1]. Complex intervention research in healthcare seeks to understand how interventions work in context and contribute to system-wide changes. This research approach generates evidence to support better decision making in clinical settings including resource-constrained healthcare settings [2]. Although the need for robust knowledge in such settings is crucial, the research itself may be a further imposition on under-resourced and over-burdened healthcare staff [3]. Our prior work indicated this might be especially true of neonatal care units in Kenya where staff, especially nurses, face huge day-to-day pressures [4–6].

## Kenyan context and NEST360 programme

Kenya is a low-middle-income country with a population of approximately fifty-two million. There are an estimated 1.5 million births per year, of which approximately 88% are facility-based, with the majority (64%) of these deliveries in public facilities [7]. Significant investments in the provision of the technologies, staff skills training and support for quality improvement for newborn units (NBU) began in 2019 through the NEST360 (Newborn Essential Solutions and Technologies) programme in partnership with the Kenyan Ministry of Health and 13 specific county hospitals [8,9]. NEST360 includes the provision of continuous positive airway pressure (CPAP) machines, oxygen concentrators, blood glucose monitors, pulse oximeters, radiant warmers, and phototherapy devices, along with associated training for health workers and local biomedical engineers. The programme has also promoted local quality improvement on the NBU including quarterly mentorship/support supervision visits. More detailed explanations of the NEST360 programme can be found elsewhere [9,10]. The 13 NEST360 sites included public hospitals already engaged in the Clinical Information Network (CIN) [11,12]. The CIN collaborates with the Ministry of Health to improve the availability and use of routine health information in Kenyan county hospitals and has supported NEST360 to provide hospitals with at least quarterly feedback reports on key NBU quality indicators [11,12]. The 13 hospitals engaged in NEST360 together admit approximately 13,000 babies per year.

## Challenges facing neonatal care in Kenya

Babies in neonatal units need close monitoring and multiple forms of treatment and support to promote survival. Low staffing levels may result in the omission of critical tasks such as vital signs monitoring and hinder effective communication across teams [13]. Equally, poor nurse-to-patient ratios are linked to missed care and higher mortality rates [13,14]. Increasing the number of skilled nurses could improve care quality by reducing missed tasks and enhancing teamwork, but evidence from studies of interventions that support this argument in low- and middle-income countries (LMIC) is lacking [15]. Nurses also often perform lower-skilled tasks, like cleaning equipment, changing linen and diapers. In many hospital settings, ward or care assistants in varied forms take on lower-skilled tasks, potentially freeing up nurses’ time to focus on skilled care and improving care quality [13,16]. Despite increased attention to improving hospital neonatal care quality, little attention has been paid to the critical role of nursing care. Beyond clinical roles, nurses and other health workers have critical roles to support families. These relationships powerfully shape whether families receive respectful and inclusive care and their wider experiences.

We designed and implemented a research programme to examine the impact of introducing new staff on care quality, staff, and family experiences within the existing NEST360 context in Kenya. In this paper, we provide an overview of 1) the intervention research design, 2) the strategy used to conduct research addressing multiple objectives and, 3) the range and extent of data generated. Finally, we reflect on learning gained through the conduct of our research. We do not report on the findings of our research here as these are reported elsewhere, and we would not be able to do them justice by attempting to report these in a single report.

## Harnessing innovation in global health to improve quality of care (HIGH-Q)

We sought to implement the Harnessing Innovation in Global Health to Improve Quality of Care (HIGH-Q) research programme in four of the hospitals that had benefitted from the NEST360 interventions. The HIGH-Q research programme had three main objectives which were:

1. To evaluate how a workforce intervention augmenting staffing numbers in four public hospitals affects:

The quality of care as measured by a nursing care index.
Staff and family experiences of care, including their relationship to the new technologies.

(2) To examine the process of post-discharge neonatal care and identify information tools (innovations) and improved care pathways might meet the needs of health workers and families to deliver higher-quality care.
(3) To examine the governance process of introducing technologies and service delivery innovations to support health (existing technologies new to the setting, novel technologies, or staff arrangements) and explore how they might be improved.

We drew on knowledge gained from previous work within this context to utilise research methods that would allow for multiple enquiries from the same data set wherever possible. Additionally, we employed regular engagement with stakeholders (clinicians, nurses, hospital managers, county health directors) to ensure we were not burdening our participants (individuals and facilities) [17]. An overall HIGH-Q study outline including the study sites, population and sample sizes is provided in Appendix 1.

## The HIGH-Q workforce intervention

Our selection of HIGH-Q intervention sites was guided by several elements, as extensively described elsewhere [18]. Briefly these include: recipients of the NEST360 programme; hospitals with active involvement in CIN quality improvement activities; and willingness and readiness to try out a supplemental workforce intervention. We deployed additional nurses into each of the four facilities’ NBUs for 15 months, expecting that by six months this extra staffing would have a measurable effect. Previous work in Kenyan neonatal units had indicated a relationship between staffing and the quality of nursing care [13]. The number of additional workforce staff we deployed was constrained by the project budget; however, we estimated that a 20–30% increase in staffing numbers would improve nursing care by at least 5%. Based on the prevailing nursing workforce establishment in the target hospitals, this translated to an addition of three nurses across each of the four units.

In this setting, registered nurses may also carry out many non-technical (non-clinical) tasks as part of their duties. We therefore further hypothesised that the addition of extra non-professional staff (i.e. ward assistants) to conduct these non-technical tasks would free up nurses’ time, enabling them to carry out core nursing tasks which are often missed. Thus, three ward assistants were introduced into the same units between months seven and fifteen of the complete workforce intervention period. In these facilities, the ward assistants hold post-secondary school education with no tertiary or clinical training.

We provide an illustration of the study settings including staffing numbers and workload before the intervention in Table 1.

**Table 1.** Study settings and context.

		Hospital			
		H1	H2	H3	H4
Location of the Hospital		Urban	Urban	Semi-urban	Semi-urban
Total Hospital Deliveries/Year*		4679	5769	3353	4384
Total NBU admissions *		1383	1705	782	1365
NBU Bed Capacity		55	60	50	38
Average bed occupancy		107%	72%	54%	50%
Number of babies on the ward (median IQR)		59 [53, 64]	43 [39, 47]	27 [23,30]	19 [15,20]
Number of nurses on the Shift (median IQR)	Day	2 [2,3]	2 [2,3]	1 [1,2]	2 [1,2]
	Night	2 [2]	3	1 [1,2]	1 [1,2]
Nurse-to-babies ratio		1:37 (34–43)	1:20 (20–23)	1:23 (17–30)	1:15 (12–18)
Total Workforce	Skill mix				
Nurses	Certificate	1 (7%)	1 (6%)	1 (17%)	0
	Diploma	8 (57%)	11 (61%)	5 (83%)	7 (58%)
	Higher Diploma (Neonatal Care)	4 (29%)	3 (17%)	0	1 (8%)
	Bachelor’s degree	1 (7%)	3 (17%)	0	4 (33%)
Paediatrician/Neonatologist	Consultant	2	2	1	2
Medical Officers (General practitioner)	Medical Officer	1	0	1	1
	Medical Officer Interns	3	2	2	1
Clinical Officers Non (Physician clinicians)	Clinical Officer Interns	1 to 5	0 to 5	2 to 5	1 to 4
Nutritionist	Nutritionist	1–2	1	1	1
Nursing Students, median [IQR]	Student nurses	2 [1–5]	7 [4–12]	7 [4–14]	6 [3–11]
Ward Assistants (WA)	WA Pre-intervention	3	3	1	2
	WA post-intervention	6	5	4	5

” >*2024 data from Kenya Health Information System (KHIS)

## The HIGH-Q conceptual framework and anticipated workforce intervention outcomes

As part of our intervention design and analytical approach, we conceptualised how the interventions would provide the desired change and some of the anticipated outcomes. This was produced through our previous experiences working within similar Kenyan NBUs, and literature on adoption and integration of new technologies into healthcare [19]. This work was conducted in a setting with poor nurse patient ratios, limited equipment availability, and poor communication between the clinical teams and parents [20]. We anticipated an increase in the nursing and ward assistant workforce would avail more time for nurses to conduct their duties, improve communication within these units, and enhance positive patient/family experiences of care.

**Figure 1.**
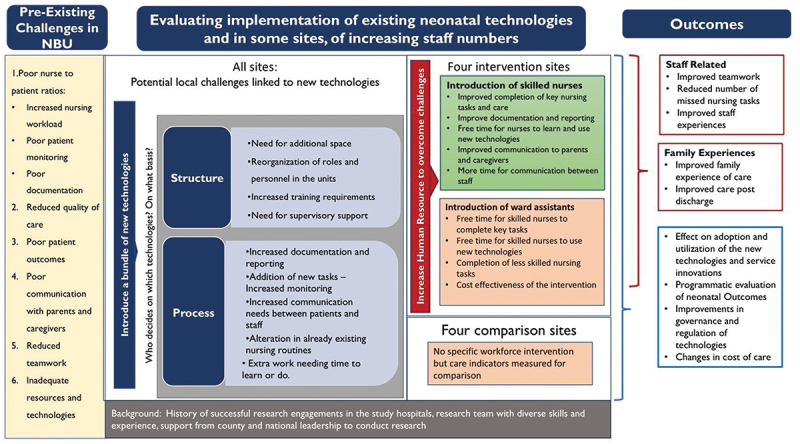
Conceptualisation of the HIGH Q intervention context.

Our main outcomes of interest after the introduction of the addition of human resources were to identify improvements in how nurses delivered care, including having a motivated team providing care with an overall improvement in the experience of mothers and caregivers (Figure 1).

## Ethical approval

Ethical approval was given by KEMRI (Kenya Medical Research Institute) Scientific and Ethical Review Committee (SERU) (KEMRI/SERU/CGMR-C/229/4203), and the Oxford Tropical Research Ethics Committee (OXTREC) (reference 26–21) and the National Commission for Science, technology & innovation (NACOSTI/P/23/27504). The study was performed in accordance with the principles stated in the Declaration of Helsinki. Informed written consent was obtained prior to data collection (observations, interviews and focus group discussions) from all the target participants. To maintain confidentiality, no personal identifiers were collected and, in the data presented here, all the study hospitals are deidentified.

## HIGH-Q data collection approach

**Figure 2.**
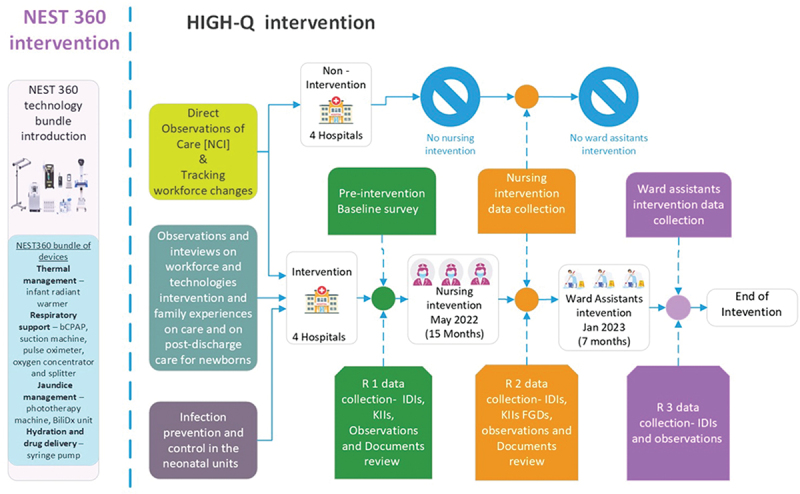
Overview of the planned research activities across the study hospitals (KII key informant interviews, FGD focus group discussion, IDI In-depth interviews).

We planned our quantitative and qualitative methods of data collection and evaluation around our staffing intervention (Figure 2) and organised our programme of research in relation to several inter-related but separate investigations (each answering a specific HIGH-Q objective/sub-objective, as outlined above).

We embedded regular stakeholder engagement in our programme of work, which included discussions with key groups prior to obtaining funding, during the entire programme of research and as part of ongoing dissemination meetings for early, interim and final outcomes. Our engagement programme, including our methods of engagement, is fully explained elsewhere [17].

Our research team in Kenya consisted of four paid research assistants and associates; these were a mix of social scientists and nurses, one programme manager, two postdoctoral research staff (paediatrician and a social scientist) and, during our structured observation periods, eight observers (one per participating hospital NBU). The programme also included five doctoral students (nurses, clinicians, and social scientists) with varying levels of involvement in on-the-ground data collection activities. The research assistants, doctoral and postdoctoral researchers had a mix of both social science and biomedical science backgrounds. The paediatricians and clinicians were responsible for the design and review of the quality-of-care data collection tools, discussing the study with the hospital clinical teams and counter checking the clinical data. Researchers with a nursing background led the nursing care and infection prevention and control observations in the study and trained the research assistants on the data collection process. The social scientists led the qualitative aspects of the study; observing what was happening in the wards, including the interactions between the mothers and nurses. They also led the interviews and focus group discussions with mothers and nurses. A table describing the researcher roles and synergies is included in the supplemental materials. Figure 2 below provides an overview of the data collection process using a mixed methods approach.

- a) Quantitative measures of nursing care and intervention effects

The primary aim of the quantitative component was to examine the pre-post study effect of adding additional nurses on care as measured by the nursing care index with the secondary aim of examining whether adding ward assistants would enable nurses to deliver more bedside care using the same nursing care index (addressing Objective 1a above). In brief, previously developed tools were employed to assess the bedside nursing care delivered to sick newborns through direct observation [21]. We aimed to observe a minimum of 220 babies per facility across the study period. Structured observations were conducted by trained medical personnel (nutritionists) over randomly selected 12-hour shifts across the study period. To allow for comparison (control), the second round was conducted in eight hospitals, including four more non-intervention hospitals (Figure 1). The study design to assess the effects of workforce intervention on the quality of nursing care is fully described elsewhere [18].

Quantitatively, data were generated across three rounds of structured observations of 12-hour nursing shifts. As mentioned, the second round was conducted in eight hospitals, including four more non-intervention hospitals. Subsequent rounds of observations were only conducted in the four intervention hospitals. 1190 babies were observed in the study (Table 2).

**Table 2.** Number of babies observed to quantitatively measure nursing care and intervention effects.

	Round 1 (Baseline)	Round 2 (Nurse intervention)	Round 3 (Ward assistant intervention)	Total
Babies observed	290	607 (300 intervention, 307 non-intervention hospitals)	293	1190
Observation Hours	3428	6548	3381	13357

b) Qualitative investigations of nursing care and intervention effects

Qualitative work focused on exploring the work of staff, (especially nurses), their use of technologies and the effects of adding new staff, and if and how concerns about new technologies and innovations are experienced, raised and responded to. Qualitative data contributed to meeting all HIGH-Q objectives. Data were gathered through interviews, focus group discussions and non-participant observations in the eight study hospitals, including the four non-intervention hospitals (Table 2).

Using multiple qualitative methods necessitated multiple observers, topic guides and interviews. This was raised early on as a concern, especially for interviews and group discussions with staff, as it could mean approaching busy and already overburdened staff to participate in several interviews covering apparently similar topics of enquiry. In response, we incorporated issues we identified *a priori*, based on literature and our prior experience, that would impact neonatal care perspectives for families and staff into our qualitative topic guides and plans for non-participatory observations [22,23]. These *a priori* topics included staff and family interactions, staff teamwork and opinions and experiences of the NEST technologies. We then identified areas where data collected under our objectives might provide useful insights for other objectives and, as a group of investigators, reviewed data collection plans and materials to ensure we could capture broad as well as rich data that would meet our multiple objectives [22,24]. For example, including questions on discharge needs (objective 2) during the interviews with mothers and nurses as part of learning on experiences (objective 1b). An additional sample of interviews investigating governance and regulation were held with higher-level stakeholders [17]. Throughout, we remained flexible to incorporate and accommodate emerging issues.

**Figure 3.**
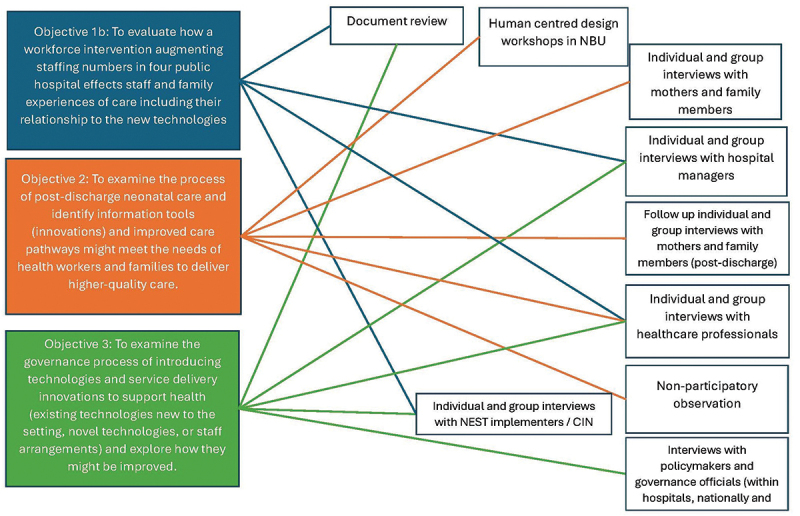
Overview of qualitative methods and objectives.

Figure 3 is an overview of qualitative methods showing synergies between our qualitative objectives and their different forms of data collection. The topic guides are included as part of the supplementary materials in this report.

## Integration/Convergence of methods and findings

HIGH-Q was a layered mixed method multidisciplinary research programme, which allowed us to examine our multiple objectives in diverse robust ways (Figure 3). While each of our objectives necessitated different methodological and theoretic approaches, our epistemological stance across the research programme was pragmatism. Taking the epistemological stance of pragmatism across the research programme gave us methodological flexibility to respond to the needs of the programme and diverse stakeholders. This stance also allowed each of our objectives to be explored using a specific epistemological lens. For example, in our first objective, our approach ranged from a positivist quantification of measures of care (i.e. objective 1a) to a phenomenological approach in our qualitative exploration of staff and families’ experiences of care in the context of introduced technologies (objective 1b).

We held ongoing meetings with stakeholders during the data collection period to ensure our data collection practices were not compromising care delivery. In one hospital, staff voiced concerns over the amount of research activity being conducted in the NBU, despite our efforts as outlined above. This was mainly due to the limited space in the NBU. In response, we held a meeting with the hospital manager to discuss how our activities could be changed or harmonised to an acceptable level. We then reduced the number of observers in the unit at any given time, the number of researchers present in an interview or group discussion, and staggered data collection so fewer researchers were in the unit at any given time. We also intentionally conducted our regular research team meetings and debriefs outside participating units/facilities.

We believe our ongoing relationship with hospital staff and management brought this issue to light early on, allowing us to continue our work with appropriate adjustments. This approach was well-received by the facility; in subsequent engagement meetings, staff expressed their gratitude, specifically mentioning reduction in the number of researchers within the unit.

Joint data collection and analysis reflective meetings among researchers across work packages provided early and ongoing understanding of our findings, which was crucial to identifying gaps in our planned data collection and adjusting our strategy to fill these gaps before the end of the study. For example, we were seeking to better understand mothers/families’ experiences of care (objective 2) and information needs (objective 3). Relevant data were gathered from one set of interviews with mothers to minimise disruption to participants and the number of interviewers needed. Emerging learning and gaps in knowledge were discussed in these meetings. Additionally, meetings allowed non-clinical research members to clarify clinical questions that emerged from observations or interviews with the research paediatricians and nurses.

## Process evaluation informed by conceptual understanding and emerging data

Process evaluation aims to systematically document a programme’s activities and how the implementors and recipients of the intervention engage with this process [25]. We planned for a process evaluation based on post-hoc analyses of the quantitative and qualitative data we expected to be available. We incorporated data from documents describing how initial plans changed with interviews and non-participant observations with programme implementers, including the nurses and ward assistants involved in the workforce interventions. However, we found in preliminary analyses that these data were insufficient for exploring specific ways that the interventions were delivered and received by staff. To augment the process evaluation, we organised additional interviews with staff (Table 3).

**Table 3.** Summary of the data collected and participant numbers in the study.

Round 1 interview						Total interviews
Objective	Cadre/level	H1	H2	H3	H4	
To evaluate how the introduction of a workforce intervention affects the quality of care (Total hours of observation = 1296)	Nurses	5	7	4	9	**25**
	Clinicians (consultants, medical officers, nutritionists, and interns	3	2	1	5	**11**
	Ward Assistants	1	2	1	1	**5**
	Mothers	0	5	6	0	**11**
	Estimated observation hours	480	240	192	384	
To examine the process of post-discharge neonatal care	Mothers IDIs	8	0	0	12	**20**
	HCWs IDIs	6	0	0	12	**18**
	Mothers FGDs	2	0	0	0	**2**
To examine the governance process of introducing technologies and service delivery innovations.	Interviews with National, County and Hospital staff					13
	FGD with NBU nurse in charges					2
	FGD with CIN pediatricians					2
	Piloting (1 hospital and 1 KPA)					4
Round 2 Interviews Post Nurses Intervention						
To evaluate how the introduction of a workforce intervention affects the quality of care (Total hours of observation = 1200)	Nurses (Non-Intervention)	5	10	5	7	27
	Nurses (Intervention)	0	0	0	0	0
	Ward Assistants (Non-Intervention)	1	0	0	0	1
	Ward Assistants (Intervention)	0	0	0	0	0
	Mothers	5	5	5	5	20
	FGDs with mothers	2	2	2	1	7
	Estimated observation hours	384	240	192	384	
To examine the process of post-discharge neonatal care	Mothers	3	10	0	0	13
	Clinicians, nurses, nutritionists, physiotherapists	0	1	0	0	1
	Mothers FGD	0	1	0	0	1
	Co-design workshops	2	0	0	0	2
To examine the governance process of introducing technologies and service delivery innovations	Interviews with National/County, NEST 360 and Hospitals staff					46
	FGD with Biomedical engineers					2
Process Evaluation 6 months evaluation	Nursing managers (non-intervention hospitals)	1	1	1	1	4
	Nurses (Intervention)	3	3	3	4	13
Process Evaluation Focus Group discussions	Stakeholder meeting (FGD)					1
	Intervention nurses (FGD)					1
Round 3 Interviews Post Ward Assistant Intervention						
To evaluate how the introduction of a workforce intervention affects the quality of care and process evaluation (12 months) (Total hours of observation = 600)	Nurses (Non-Intervention)	4	5	5	7	21
	Nurses (Intervention)	3	3	3	2	11
	Ward Assistants (Non-Intervention)	2	2	1	1	6
	Ward Assistants (Intervention)	3	3	3	3	12
	Estimated observation hours	150	150	150	150	

## Discussion

Our study planned to assess the effects of neonatal quality of care resulting from employing extra nurses and ward assistants while hospitals introduced a bundle of technologies and to conduct our research without placing undue pressure on healthcare workers and carers. This work was participatory and pragmatic by nature, and thus we applied our emerging learning within study hospitals as part of our ongoing engagement to improve care in neonatal units. Sharing our emerging results and critical concerns with hospitals and health leadership teams, we hoped would contribute to high-quality, safe care being provided in Kenyan hospitals [26]. We aim to use our research outcomes to identify where systems of care can be improved at county and national levels, with a particular focus on issues of neonatal unit staffing, to help policymakers and planners address critical needs promptly.

We were cognisant when designing our study that there might be unintended negative consequences of introducing new staff into some facilities, and that during data collection, researchers might observe situations of ethical concern [27]. In response, we designed new staff job descriptions together with regional, national, and international stakeholders to specifically ensure their roles, qualifications, training and reporting structures were clear to minimise role creep. We also anticipated that health facilities grappling with significant staff shortages might decide to redeploy new staff to other units. To mitigate this, we signed agreements with these facilities to limit redeployments.

Other areas where codesign was crucial were in the design of new post-discharge tools (objective 3). Additionally, we sought scientific and ethical approval of all activities, and incorporated specific ethics debriefs into our regular team meetings. We sought to ensure our research activities did not hamper service provision in facilities by working with staff to find appropriate opportunities for interviews and conversations and limiting the number of researchers present at any given time. When issues emerged, we responded quickly to protect clinical care, and preserve our relationships with stakeholders and programme objectives.

The ethics of implementing a workforce intervention and then withdrawing these staff were considered from the outset. Appreciating the nature of research and resource limitations, we engaged potential study hospitals and the departments of health before research began to explore ways that these staff members might be assimilated into the hospital workforce after the project. In two of the hospitals, some nurses were employed by the hospital after the study period.

HIGH-Q’s multifaceted approach allowed us to examine the quality of neonatal care comprehensively spanning aspects of human resources, service delivery, leadership and governance, and integration of medical technologies [28]. Using a flexible, iterative approach allowed us to investigate these multiple factors in resource-constrained neonatal settings without placing undue pressure on already-stretched facilities and staff. Through a team-based approach to data collection tool creation, regular debriefings, and analysis meetings, we identified research synergies and where we needed to alter or expand our planned activities.

We believe that taking an overarching pragmatic epistemological stance in our research programme allowed for our multiple objectives to be explored in diverse ways. Each objective was a sub-study, using appropriate methodological, theoretical and analytical approaches to meet that specific objective (e.g. as seen in our reported findings) [29–31]. Pragmatism helped to bridge the gap between individual objectives, and between the need for scientific rigour and practical ethical responses to stakeholder and participant needs inherent in implementing a complex, multi-objective research programme in resource constrained settings.

### Limitations and delimitations of our approach

As is the case with observational studies, it was anticipated that study participants would change their behaviours knowing they are being observed (Hawthorne effect). While we acknowledge this limitation in our intervention evaluation, our researchers spent a week in the neonatal units to familiarise themselves with routines, shifts, and staffing before gathering any data. This helped build rapport with staff, facilitated informed consent, allowed questions about the study, and surfaced potential concerns from staff and caregivers. Additionally, some research team members had worked in the study hospitals previously and were known to the staff members. We believe these steps reduced the Hawthorne effect.

A challenge with interviews and observations in hospitals is potential for moral distress that may be experienced by the research staff stationed in the neonatal units, which might manifest differently for those with and without clinical training. To support researchers, we organised regular debriefing sessions during data collection, with careful response processes to issues raised built into the overall interactive approach.

Although our layered mixed methods allowed us to examine multiple objectives in diverse robust and efficient ways with limited disruption, this was not always the case. Sometimes data collection (e.g. interviews) had to be disrupted or rescheduled with patient care taking necessary precedence. Additionally, our team meetings elucidated additional data collection needs (e.g. repeat interviews with healthcare staff for our process evaluation). These repeat interviews, although few, were not always taken positively by some participants.

A limitation of replicating our approach in other settings is that we required a large research team with diverse skills, all of whom were trained not only in their data collection methods and the overall programme objectives (as is standard practice) but also in understanding methodological techniques unfamiliar to them linked to associated objectives. This meant that the core research team attended training and ongoing activities such as team debriefings and mixed method workshops (for example, the creation of the conceptual models and plans for process evaluations). This required a dedicated, well-funded, and active training package. Creating acceptable and feasible plans for our data collection within facilities and gaining access to governance and regulatory stakeholders also required a funded and active stakeholder engagement work package [17]. We were fortunate that our funders provided this and recommend that all funders allow for – and encourage – these aspects when considering investment in large, multi-faceted research programmes.

## Conclusions

This paper reports our experience and reflections on the practicalities of the design and evaluation of a workforce intervention in the context of other care improvement interventions, including acting on feedback from the stakeholders during the study.

Regular engagement with county and hospital leadership and staff on the ground led us to refine our research methods and implementation plans and supported the collection of a large multi-faceted dataset with minimal staff burden. We recommend that researchers employ stakeholder engagement and carefully consider the impact of data collection methods within large multi-faceted research studies in busy low-resource health settings.

We highlight that prior experience and consultations with stakeholders alongside our pragmatic epistemological approach meant we could streamline our data collection activities and explore multiple research questions and objectives. In HIGH-Q, we were able to conduct good quality multi-disciplinary work balancing field realities where there is an ethical and pragmatic imperative to support stakeholder needs, and academic scientific quality. We believe that our use of the pragmatic epistemological lens contributed to our flexibility and could be considered as a way through this common difficulty.

## Supplementary Material

SQUIRE2ChecklistHIGHQ.docx

HIGHQMethods Supplementarymaterials.docx

## Data Availability

The data used for this report is available upon request. Applications for access can be made through the Data Governance Committee with details available on www.kemri-wellcome.org, or by email to **cgmrc@kemri-wellcome.org**.

## Preprint

This paper is available on SSRN as a preprint.

M. Maina, S. Molyneux, F. N. Were, D. Oluoch, E. Mutua, D. Gathara, et al. Applying a Multi-Layered, Mixed Methods Approach to Evaluate Technology and Workforce Interventions in Kenyan Neonatal Units SSRN. 2025. doi: http://dx.doi.org/10.2139/ssrn.5230415
