# Supplementary material for: Applying a multi-layered, mixed methods approach to evaluate technology and workforce interventions in Kenyan neonatal units
Source: Glob Health Action. 2025 Sep 25;18(1):2558267. doi: 10.1080/16549716.2025.2558267 (PMC12487688; doi:10.1080/16549716.2025.2558267)
Supplement: HIGHQMethods Supplementarymaterials.docx [file ZGHA_A_2558267_SM9896.docx]

**Appendix 1: Study Methods Overview**

| Objective | Study (design) | Sites  (Hospitals) | Population | Sample size | Frequency |
| --- | --- | --- | --- | --- | --- |
| Objective 1a: determine the effects of new technologies on a set of quality-of-care indicators in 14 hospitals implementing a bundle of technologies (the NEST bundle). | | | | | |
| 1a | Before and after | 14 | Babies admitted to NBU | 1182 babies (591 before and after) | Long-term tracking of Quality-of-care indicators using routine data |
| 1 a | Case audits | 14 | Babies on CPAP | 130 babies on CPAP |  |
| Objective 1b: examine the programmatic approach to and process of delivering and implementing these technologies to identify factors enhancing or constraining their uptake. | | | | | |
| 1b | Document review.  In-depth Interviews (IDIs) | Four hospitals implementing NEST 360.  (two well-performing two poorly performing) | NEST program leaders, trainers, mentors, supporting biomedical engineers.  Hospital managers | 3-5 interviews with NEST / CIN staff in each of the four hospitals (12-15 total).  3-5 interviews with hospital staff in each of the four hospitals (12-15 total) | Throughout the NEST implementation in hospitals |
| Objective 2a: determine the effects of a workforce intervention on the quality of care as measured by a nursing care index in four hospitals. | | | | | |
| 2a | Before and after study with repeated cross-sectional observations | Four (not the same hospitals as in 1b) | Babies in NBU | 426 babies (213 before and after) | - Baseline - At 8 months (Introduction of nurses) - At 15 months (Nurses + Ward assistants) |
| Objective 2b: explore how the workforce intervention introduction/implementation affects staff and family experiences of care, including their relationship to the new technologies. | | | | | |
| 2 b | Non-participatory observations  IDIs  GIs | Four workforce intervention hospitals (not the same hospitals as in 1b) | Mothers, family members, Nurses, paediatricians, ward assistants | Universe of staff and mothers in the four neonatal units (non-participatory observation)  5 – 8 IDIs with staff in each hospital  2 – 3 GIs with staff in each hospital  5 – 8 IDIs with mothers per hospital (post-discharge) per data collection round  Three families per hospital (post-discharge) per data collection round (GI) | - Baseline - At 7 - 9 months (Introduction of nurses) - At 14 -16 months (Nurses + Ward assistants) - Baseline - At 7 - 9 months (Introduction of nurses) - At 14 -16 months (Nurses + Ward assistants)   Staff GIs at baseline and post-nurse and Nurse + ward assistant interventions.  2-3 weeks after discharge from hospital |
| Objective 3: examine the process of post-discharge neonatal care and identify how improved care pathways and information tools might meet the needs of health workers and families to deliver higher-quality care. | | | | | |
| 3 | Non-participatory observations  IDIs  GIs  Participatory design workshops | Four (one urban, one rural hospital and two community clinics) | mothers, family members, hospital staff, community workers | A maximum of 78 participants   - Up to 10 IDIs with out-patient paediatric/neonatal and MCH clinic staff - IDIs and non-participatory observation with up to 10 mothers/families in paediatric/neonatal and MCH clinics - Two GIs (6-8 participants each) - Eight participatory design workshops in 2 hospitals and 2 community settings with 6-8 participants each | - After the introduction of the workforce interventions - From 2 months to 6 months after discharge from hospital |
| Objective 4: examine the governance process of introducing technologies and service delivery innovations that aim to support health (either existing technologies new to the setting or novel technologies or staff arrangements) and explore how they might be improved | | | | | |
| 4 | Document review (from 1b, and notes from regular meetings and debriefs)  IDIs | 14 | Regulatory and governance stakeholders (from ethics review boards, regulatory bodies, Ministry of Health, international bodies, e.g., WHO and UNICEF)  Senior researchers/ implementers of the NEST program and CIN  Hospital managers  Frontline health workers | 10-20 regulatory and governance stakeholder interviews  12-15 interviews with senior researchers and implementers from NEST and CIN (reanalysis of data from objective 1b)  12-15 interviews with NBU managers (reanalysis of data from objective 1b)  12-15 interviews with healthcare workers (reanalysis of data from objective 2b) |  |
| Objective 5: determine the costs and budget impact of introducing interventions (technologies and additional workforce) in newborn units. | | | | | |
| 5 | Document review.  IDIs | Workforce  Four hospitals | Medical record review of babies in NBU  Hospital administrative records | 426 babies (213 before and after) |  |
|  |  | Technologies  14 hospitals | NEST program leaders, trainers, mentors, supporting biomedical engineers)  Hospital staff (ward managers, nurses, biomedical engineers, paediatricians) | 4 -5 interviews with program staff  10-20 interviews with NBU staff (including managers) and hospital managers | Throughout the NEST implementation in hospitals |

**Appendix 2:** **Interview guide for NBU nurses after the introduction of ward assistants**

1. How has your experience been since the introduction of the new ward assistants?

- Impact on your workload job satisfaction, rest and leave days, management of babies, task completion).
- Perception of work done by ward assistants, is it adding to their work or have they lessened the burden on the nurses)
- Are there things that nurses have or are still training the new ward assistants on? (Probe is on skill demonstration)
- In relation to your tasks/roles on the NBU, are there things that nurses still struggle with

1. What non nursing tasks have you been able to shift to the ward assistants.

- From your experience so far, how would you comment about their ability to take on the delegated tasks (competency, need for supervision)

1. Have there been changes in the tasks performed by nurses (Probe whether there are non-nursing tasks being performed by nurses and why)
2. Have there been issues that have affected their performance or ability to accomplish their tasks, if so which ones?

- Distribution of ward assistants per shift both day and night, weekdays and weekends
- Retention, redeployment or WA’s serving NBU and other wards

1. Looking at the tasks performed by ward assistants, are there tasks that they find easy to perform or tasks that they struggle with (Probe how nurses support with the difficult tasks)
2. Are there tasks that ward assistants and nurses perform together? (Probe is on task-sharing)
3. How would you describe your relationship with the new ward assistants? (Probe on;

- Relationships across cadres (WA’s and nurses, other cadres in the NBU)

1. With the introduction of ward assistants, how would you describe the relationship among nurses, and other cadres in the NBU? (probe is on nurse-nurse relationship, nurse-other cadres’ relationships)
2. With the new assistant ward, have there been changes in terms of the support offered to mothers in the NBU? (Probe for,
   - How WAs support mothers and perceived effect on mothers’ experiences.
   - Has the introduction of the ward assistants changed the way nurses support mothers/ interaction with mothers and perceived effect of this on mothers’ experiences?
3. Are there things that mothers still struggle with? (Probe whether there are any challenges and ways to better support mothers)
4. As we conclude, what recommendations would you make in terms of better integration/performance of ward assistants.

**Appendix 3: Observation guide exploring the impact of ward assistants (workforce intervention) on the day-to-day running of newborn units across the 4 HIGH-Q sites.**

**Observe:**

**Context**

- Number of ward assistants in the newborn unit. (Total number of Non-HIGH-Q and HIGH-Q ward assistants)
- Nature of employment (Permanent, casual, contract, facility arrangement, outsourcing and the impact on work)
- Distribution of ward assistant per shift (day and night, mixture of new and resident ward assistants in a shift, are there changes in the allocation of shifts between the HIGH-Q and non-HIGH Q ward assistant)
- Allocation of tasks outside the NBU or redeployment to other departments (for Non-HIGH-Q and HIGH-Q ward assistants)
- Supervision (Who and how supervision is done, are there differences in supervision between the HIGH-Q and Non-HIGH-Q ward assistants?)
- What is the ‘mood’ of the place? Tensions

**Roles/tasks performed**

- What tasks are performed by who, are there differences in tasks performed by the HIGH-Q and non-HIGH Q ward assistants)
- Are there non-nursing tasks that are still being performed by nurses?
- How nurses are utilizing time that they should have been doing non nursing tasks
- Task sharing, task completion, task shifting, job satisfaction. (Are there differences between Non -HIGH-Q and HIGH-Q ward assistants) are how the WAs think they are helping nurses, do they feel they are making nurses’ work less stressful, are they helping ‘share the burden’ etc? Or do they see nurses retreating to do the paperwork or taking longer breaks?

**Relationships**

How are the HIGH-Q ward assistants interacting with?

- Non-HIGH-Q ward assistants
- Nurses including the nurse-in-charge
- Mothers
- Other cadre of staff in the NBU (consultants, student nurses, COs and MO interns)

What do they talk about with?

- Non-HIGH-Q ward assistants
- Nurses including the nurse-in-charge
- Mothers Do they have problems with the parents?
- Other cadres in the NBU (consultants, student nurses, COs and MO interns)

**Support**

- How are ward assistants supporting mothers? (Observe support provided, timeliness of support and the impact on mothers and family experience)
- Are there differences in support provided by the HIGH-Q and non-HIGH Q ward assistants?
- Capture from informal conversation the perception of mothers on ward assistants)
- Observe mother’s needs (Have there been any changes in mother’s needs) Do they think they are a help to the parents, in what ways?
- **Successes and struggles**
- Are there tasks that ward assistants find easy to perform? (observe both HIGH-Q and non-HIGH Q ward assistants)
- Are there tasks that ward assistants are struggling with? (Observe common challenges among both HIGH-Q and non-HIGH Q ward assistants, differences if any between the HIGH-Q and non-HIGH Q ward assistants)
- How do the ward assistants deal with their struggles?

1. **Appendix 4 Interview guide for high-level national and county stakeholders.**

❖ **Please, can you describe your professional role.**

How are you or your office involved in medical device regulation, management and oversight?

❖ How are medical devices approved or regulated in your agency?

- What guidelines are available and followed? E.g policies, essential lists.

Ask around requirements such as testing and evaluation, use of standards, import permits and controls, registration of vendors, and post marketing surveillance.

• What are the key considerations for certification and approval of medical devices in Kenya.

Are issues of quality, safety considered, and how is this ascertained.

• Are there some medical devices or technologies that do not go through an approval process, or other special circumstances?

• Is there recognition of international regulations, recommendations, and certification?

❖ How are these regulations enforced? What has worked well? What are the challenges with enforcement (funding, training, infrastructure, personnel?)

- What are incentives and disincentives for monitoring and oversight?
- Are there other challenges with implementing regulatory guidelines?
- Are there alternative entry points for potentially poor quality or unsafe medical devices?
- Is there training for regulatory officers to perform duties, who organises this. What does this training involve?
- Is there adequate staffing to carry out regulatory and oversight functions?
- Does regular training and capacity building occur?

❖ Can you describe the processes after approval has been given for a medical device or technology?

- Is there follow up after approval to facilities to monitor devices.
- How does the organisation work with counties and facilities, if at all
- What other agencies are involved in medical device regulation and oversight? Are there any overlaps and tensions between these agencies and yours?

❖ Do you have any feedback reports/systems to know how oversight is working in practice in counties and hospitals? If not, what feedback do you think is helpful to you?

❖ Please give us your opinion on the certification and oversight of medical devices in Kenya? What are some points in the approval process that things go well and points where things do not go well?

1. Can you describe planning for procurement and supply for medical devices?
2. What key stakeholders are involved in planning for procurement and supply of medical devices?
3. What activities or interactions are involved in this process? Methods, approaches.
4. What factors are considered in procurement planning?
5. What regulatory and oversight concerns are considered in procurement processes?
6. What might be challenges with medical device regulation and oversight?
7. Please can you describe the process of introducing medical devices through the county?
8. Are there any national level processes and considerations? Such as working with KEMSA, PPB and their county equivalents?
9. Are there differences in the processes between donated devices and devices procured by the county?
10. What is the process for devices/equipment brought in by national government e.g. through the MES program, does this work differently?
11. Have you heard of or been involved with the NEST program bringing in newborn devices? Has this program been different from others? If yes, how so.
12. What other donors has the county worked with to bring in medical devices? What has worked well and what might be some challenges with working with donors?
13. What interactions happen with facilities and how are the needs at facility level included or communicated?

❖ Do you have anything else you would like to add and are there questions you have for me.

**Appendix 5 HIGH Q Interview guide for Health Facility level actors**

Period worked on in hospital:

**Introduction of medical devices/technologies:**

1. Can you describe your role in the hospital, and how it related to selection and introduction of medical devices.
2. What are the processes for introducing technologies?
3. Who is involved in this process? Actors, committees? Who approves and what lines of reporting are required.
4. Are medical doctors/paediatricians/nurses involved in the selection and prioritisation of medical devices/technologies introduced into the hospital? How does this process happen?
5. Are these health workers (nurses, doctors) part of any hospital committees for medical devices?
6. What are the key considerations/criteria taken for selection, prioritisation, and approval?
7. Are there instances where other factors influence the selection/approval of medical devices or equipment?
8. Are there differences between processes for medical technologies brought in through routine procurement and those donated to the hospital.
9. Are you familiar with the NEST 360 program work introducing newborn technologies? Can you describe the processes for introducing these technologies. How does this differ from other donations?
10. **Training**: Is there provision made or put in place to ensure staff get the required training to use technologies when procured or donated?
11. Who makes such training arrangements, and which staff are usually trained? (Hospital, donor, budgeted for routinely).
12. Is there a training plan/schedule in place and how is training assessed if at all?
13. What has worked well in terms of training and what are some challenges?

**Selection and procurement processes**

- How are the devices and their supporting supplies (consumables, spare parts, replacements) procured and supplied?
- What key actors/teams/committees are involved and what activities or interactions happen to reach decisions on procurement?
- What are the approaches taken to inform procurement planning? E.g are the NBU needs considered, use of past experiences/suppliers?
- What other factors are considered? E.g regulatory approval, cost, specialist recommendation, guidelines etcs
- Can you reject certain devices donated, procured or supplied? What might be the reasons and what would that entail?
- What has worked well and what are some challenges with supply and procurement processes?

**Monitoring and oversight:**

- How are devices monitored and overseen after introduction? Are there systems to ensure devices are used properly? Who provides supervision while devices are in use?
- Are staff trained to monitor equipment, identify and raise concerns? Who is trained and who is not?
- How do you handle reporting of adverse events and complications or problems with devices observed during clinical practice? Who are these problems reported to? What do you do with this information?
- Have you had any concerns about how the newborn devices? Can you give examples of such concerns or problems?
- How do you document and share info on device problems or other concerns related to devices? What policies or mechanisms are in place? With whom do you share these concerns?
- Do devices raise additional requirements for monitoring and oversight?

**Maintenance and Management: How are devices managed and maintained:**

- Who handles and maintains inventory? Are there systems in place for preventive maintenance and repair? Who does it and how is this recorded and ensured?
- Are there systems for logging device problems and malfunction, system for communicating with biomed and recording actions?
- Are there any service agreements or contracts set up to maintain equipment? Does this differ for donated, procured devices? and NEST equipment?
- What factors influence maintenance and management of the devices? Funding, turnaround time for service, training for biomed etc
- How are devices with problems or non-functional devices withdrawn? What processes are involved and who makes the decision?

Is there anything else you would like to add on these topics? Do you have any questions for me?

**Appendix 6 HIGH Q Research Role Distribution and Synergies**

| **Objective** | **Primary researcher Included and key roles** | **Supporting researcher and roles** | **Overarching support** |
| --- | --- | --- | --- |
| 1.To evaluate how a workforce intervention augmenting staffing numbers in four public hospitals affects |  |  |  |
| a) The quality of care as measured by a nursing care index. | Paediatricians (training data collection, engagement with hospital managers and research monitoring)  Nurses (Training and research monitoring | Nutrtionists and nurses (data collection on quality of care) | Project Manager (Engagement) |
| b) Staff and family experiences of care, including their relationship to the new technologies. | Social Scientists (observations, Interviews and Focus group discussions) | Research Nurses (clarification of any clinical queries from the observers) | Project Manager (Engagement) |
| 2.To examine the process of post-discharge neonatal care and identify information tools (innovations) and improved care pathways might meet the needs of health workers and families to deliver higher-quality care. | Social Scientist (Observation, interviews, human centred design workshops) | Nurses and Paediatrician (clarification of medical terms and review of discharge documents and support in the workshops | Project Manager (Engagement) |
| 3.To examine the governance process of introducing technologies and service delivery innovations to support health (existing technologies new to the setting, novel technologies, or staff arrangements) and explore how they might be improved. | Social Scientist (interviews) | Project manager (facilitate the engagement with hospital leaders, ministry of health and regional leaders | Project Manager (Engagement) |
